# Mental Health and Suicide Research with Migrants in Australia: Necessary Knowledge, Skills and Engagement Strategies

**DOI:** 10.3390/bs15050604

**Published:** 2025-05-01

**Authors:** Harry Minas, Dominic Diocera, Erminia Colucci

**Affiliations:** 1 Global and Cultural Mental Health Unit, Centre for Mental Health and Community Wellbeing, School of Population and Global Health, The University of Melbourne, Parkville, VIC 3010, Australia; 2 Grampians Public Health Unit, Grampians Health, Ballarat Central, VIC 3350, Australia; dominic.diocera@gh.org.au; 3 Department of Psychology, Faculty of Science and Technology, Middlesex University, London NW4 4BT, UK; e.colucci@mdx.ac.uk

**Keywords:** mental health, suicide, migrants, refugees, research, methods, lived experience

## Abstract

(1) Research is fundamentally important in developing evidence-informed and effective policies and appropriate programs and services to reduce the burden of mental health problems, and prevent suicide, among migrants. However, this population continues to be underrepresented in mental health and suicide research, resulting in large evidence gaps that limit policy making, service design and delivery, as well as evaluation of outcomes. (2) Experts in mental health and suicide prevention research with migrants provided free-text responses to a survey that asked about the knowledge and skills required to effectively conduct mental health and suicide prevention research with migrants, and effective strategies for engaging migrant and refugee communities in such research. An adapted thematic analysis method was used to analyze the free-text responses to the six questions. (3) The study identified specific areas of knowledge and skills required for effective mental health and suicide research with migrants; the methodological and ethical challenges that may arise in such research; and strategies that are likely to be effective in engaging people with lived experience and migrant and refugee communities in such research. (4) The findings from this project can be used to inform researchers on how to ethically and effectively undertake mental health and suicide research with migrant and refugee populations.

## 1. Introduction

Over the past five decades, there has been a substantial increase in the number of global migrants, including voluntary migrants, asylum seekers and refugees, constituting 3.6% of the global population (McAuliffe & Oucho, 2024). In countries with active migration programs, such as Australia, the increase has been much larger, with migrants constituting 29.9% of the Australian population in 2020. In 2023, migrants (including refugees and asylum seekers) and their Australian-born children constituted almost 50% of the Australian population (Table 1). Migrants came from more than 250 countries and territories, (Australian Bureau of Statistics, 2024) bringing with them a remarkable diversity of languages, religions, cultures and experiences. The ten most common countries of birth of Australian migrants in 2023 were England, China, India, New Zealand, Philippines, Vietnam, South Africa, Malaysia, Nepal and Italy. The overall ratio of males to females for the Australia-born and overseas-born is approximately equal (100 and 96, respectively), while the overall median age of Australian-born and migrants (35 and 43 years, respectively) is higher in the overseas-born (Australian Bureau of Statistics, 2024).

The variety of languages, cultures and experiences of migrants—in their countries of origin, during the migration journey and during the process of settlement and acculturation in Australia and other host countries—result in equally diverse mental health needs (World Health Organization, 2023b). Despite the heterogeneous findings in the mental health literature, and the frequent finding that migrants tend to be healthier than the host country population in the period following migration, studies suggest that this generally better physical and mental health is not maintained in the long term, and that migrants overall are at increased risk of developing mental disorders and suicidal ideation (Anderson et al., 2017; Chen et al., 2019; Donath et al., 2019; Farooq et al., 2021; Hasan et al., 2021).

The difficulties encountered in the migration process by refugees are substantially different to those experienced by economic and other migrants. Although many refugees demonstrate remarkable resilience despite having experienced difficult and often traumatic circumstances, refugees overall are consistently found to have a higher prevalence of mental disorders than other categories of migrants (Smyth et al., 2023). They have frequently experienced exposure to multiple types of threats, physical and psychological violence and other human rights violations. For asylum seekers, prolonged uncertainty concerning their status in the country to which they have migrated is a very common problem. Circumstances such as these are known to have profoundly negative mental health impacts (Coffey et al., 2010; Dapunt et al., 2017; Momartin et al., 2003; Nickerson et al., 2011; Silove et al., 2007; Smyth et al., 2023; Steel et al., 2002, 2006).

The risk of suicide is highly variable among migrant groups (Donath et al., 2019; Farooq et al., 2021; Hedrick & Borschmann, 2023; Maheen et al., 2024; Pham et al., 2023a, 2023b; Plener et al., 2015; Spallek et al., 2015; Tham et al., 2023) with some evidence that suicidal behaviors may be more common among the children of migrants. The impact of adverse migration experiences can be more severe and long-lasting among children, who represent a substantial proportion of asylum seeker and refugee populations (El Baba & Colucci, 2017).

While the mental health of migrants has received increased attention in recent years (Abubakar et al., 2018), people from migrant backgrounds continue to be underrepresented in mental health, suicide and general health research (Garrett et al., 2010; Minas et al., 2013), resulting in major evidence gaps in all relevant research domains—prevalence, determinants, conceptions of mental health and illness, patterns of service use and the design and delivery of mental health policies, programs and services (Minas et al., 2013). This finding has been echoed in two recent reports from the World Health Organization (World Health Organization, 2023a, 2023b), which suggest that the insufficient data and evidence on mental health of migrants has hindered the development and implementation of legal frameworks and policies and mental health services that address the needs of these populations and has contributed to perpetuation of mental health inequities. “*More high-quality research on health, migration and displacement is crucial to developing effective policies and actions for the WHO Triple Billion Targets: advancing universal health coverage (UHC), addressing health emergencies and promoting healthier populations. Persistent research gaps in these areas greatly impact the health of people who have migrated or been forcibly displaced and the health of communities worldwide. These gaps also jeopardize the attainment of the Sustainable Development Goals (SDGs).*” (World Health Organization, 2023a).

Research to generate the required evidence is a fundamentally important contributor to the development of effective policies and appropriate services to reduce the burden of mental health problems and suicide among people from migrant and refugee backgrounds in receiving countries (Kirmayer, 2012; Whaley & Davis, 2007) to reduce barriers to mental health service access (Alshamary et al., 2024; Colucci et al., 2015, 2017b; Lee et al., 2014; Salami et al., 2019, 2020; Schouler-Ocak et al., 2024) and to improve mental health and suicide prevention outcomes (Kisely et al., 2021; Waxmann et al., 2022). Also, it is increasingly recognized that the full participation of people with lived experience and carers (PWLE), and their communities, in decision-making about all aspects of mental health programs and services, and research, is essential (Romios et al., 2008; State of Victoria, 2021).

Despite these observations, barriers and challenges exist in effectively and ethically engaging PWLE from migrant or refugee backgrounds in service planning and research (Enticott et al., 2017; Gabriel et al., 2017). These include poor representation, difficulties in communication and unfamiliarity with research among PWLE from migrant and refugee backgrounds (Oliver et al., 2004). These challenges can be addressed by employing appropriate skills and methods and allocating necessary resources to effectively and productively harness and maximize the involvement of PWLE (Oliver et al., 2004). However, there is limited information about what specific research knowledge and skills are required to do effective and inclusive mental health and suicide research with migrant communities, and what approaches to engagement of the communities in such research are likely to be most effective.

The objective of this study was to investigate the knowledge and skills required by mental health and suicide researchers, the methodological and ethical challenges that frequently arise in research with these communities, and the strategies that can assist in engaging migrants with lived experience of mental illness, their families and carers, and migrant communities, in mental health and suicide research.

## 2. Methods

This research was undertaken as a component of a larger study (Colucci et al., 2017a) to develop a mental health and suicide research agenda for people from migrant backgrounds. In that study, policy makers, service providers, academics and PWLE with expertise in mental health and/or suicide of immigrants and refugees were invited to form the expert panel and participate in the study. Potential participants were identified through a search of the relevant literature and websites, key Australian mental health or suicide prevention organizations and multicultural mental health networks, as well potential participants identified via snowball sampling and individuals who self-nominated after seeing the study advertisements being invited to participate. In addition, two consultations were held with service users and people working with service users and carers to facilitate the recruitment of people with lived experience. Delphi consensus methods (Minas & Jorm, 2010), which involved consulting a panel of experts through a series of surveys, were used to create a mental health and suicide research agenda (Colucci et al., 2017a).

In the first survey round of the research agenda study surveys, participants who identified as having expertise and experience in undertaking mental health and/or suicide research about or with people from migrant and refugee backgrounds were invited to provide up to three free-text responses to each of the following six questions:

1. Please list particular research skills and competencies required for doing mental health and suicide research about/with people from migrant and refugee backgrounds.
2. Please list particular knowledge required for doing mental health and suicide research about/with people from migrant and refugee backgrounds.
3. In your experience, what are the main methodological and practical challenges in doing mental health and suicide research about/with people from migrant and refugee backgrounds?
4. In your experience, what are the main ethical challenges in doing mental health and suicide research about/with people from migrant and refugee backgrounds?
5. In your experience, what are the most important strategies for engaging carers and consumers (PWLE) of migrant and refugee background in mental health and suicide research as collaborators and/or participants?
6. In your experience, what are the most effective strategies in engaging the broader community, particularly migrant and refugee communities, in mental health and suicide research knowledge exchange/translation (such as communicating and/or implementing research findings)?

A thematic analysis method was used to analyze the free-text responses to the above questions (Braun & Clarke, 2006). The analysis used an inductive ‘bottom up’ approach in which themes emerged from the data without the use of an *a priori* coding frame other than the frame produced by the questions that were asked. Themes were generated from the semantic and inferred meanings of the participants’ statements. Codes were created in an iterative way until no further themes emerged (code and theme saturation). Individual statements were then allocated to the derived summary themes. The analysis involved the following steps: (1) familiarization with the data through repeat reading; (2) generation of initial codes; (3) searching for themes; (4) reviewing themes; (5) defining and naming themes. The final step, number 6, in Braun and Clarke’s recommendations for thematic analysis (Braun & Clarke, 2006), is the production of this report.

## 3. Results

### 3.1. Participants

A total of 138 experts took part in the larger research agenda study. Among the participants were PWLE. Of the 138 participants in the research agenda study, 31 respondents identified as having relevant research expertise and experience and participated in this component of the project (Table 2) and provided up to three free-text responses to each of the six questions. None of the participants with lived experience in the larger study identified as having relevant research expertise and experience, which was an inclusion criterion for this component of the study.

As shown in Table 2, the respondents who had experience in conducting research about or with people from migrant and refugee backgrounds were mainly from mental health and suicide prevention services. This was followed by respondents working in universities or other research centers. Most of the respondents were psychologists/counsellors, nurses, and social workers. In total, 20 (64.5%) of the 31 experts who completed the survey questionnaire were female. While 16 (52.0%) were born in Australia, the other 15 (48.0%) were migrants, born in Brazil, China, Colombia, England, France, Greece, India, Italy, Scotland, Somalia, South Africa, South Sudan, the UK and the USA. The mean age of the panel members was 46.9 years.

### 3.2. Emergent Themes

The key themes that emerged from respondents’ responses to the six questions, the total number of responses to each question and the frequency with which themes were mentioned are summarized in Table 3.

### 3.3. Required Research Skills

*Engagement skills:* The most frequently mentioned skill was the capacity for respectful engagement with communities and research participants, without which other important skills cannot be exercised. It was noted that researchers should display qualities such as cultural sensitivity, humility, patience, flexibility and respect for other cultures.

*Research skills, experience and qualifications:* For mental health and suicide research technical research skills—such as research design, using appropriate sampling methods for representative samples, data management and data analytic skills—were all emphasized, as was cultural competence. In considering research designs, it was suggested that researchers should be proficient in employing varied and multiple methods of research—including participatory and action research—to capture the complexity of issues relevant to migrants. It was also considered important that, as well as having the necessary research qualifications and skills, researchers should also have experience in working with migrant and refugee communities.

*Communication skills:* High-level communication skills were seen by some participants as essential. Being fluently bilingual and being able to speak the primary language of research participants was identified as an invaluable skill. In the absence of this, having the skills to work effectively with interpreters was seen as essential.

### 3.4. Required Knowledge

*Knowledge of context:* A key area of required knowledge that was identified was knowledge of the context of the community with which the research is being undertaken. This includes the community’s history, language/s, social and cultural norms and community structures and dynamics. Moreover, researchers should understand the differences in the cultural and migration experiences and expectations of migrants, including an understanding of the role of cultural and social elements such as spirituality and religion in mental health and suicide. This also includes the different experiences of resettlement and acculturation. Survey respondents also noted that researchers should know about immigration and refugee policies, successful health interventions and models of mental health and suicide, and current debates in the literature. An understanding of context also includes circumstances in the country of origin, particularly for refugees when these circumstances are a cause for concern for families and friends.

*Research knowledge:* Specific research knowledge that was highlighted was an understanding of how culture relates to mental health and suicide, how mental health issues and suicide are perceived by the particular migrants participating in the research and how social and cultural factors in the country of resettlement have an impact on mental health and on help-seeking decisions. An awareness by researchers of their own culture and how this influences their own views on mental health and illness, and on the community with which they are working, was noted as necessary knowledge.

*Communication:* As in the responses to Question 1 which was focused on communication skills, knowledge of relevant communication matters was highlighted in response to this question.

### 3.5. Methodological and Practical Challenges

*Values and beliefs and language difficulties:* Understanding the cultures and values of research participants and their communities and difficulties in communication due to participants’ limited English fluency, because of the use of technical language by researchers that is unfamiliar to participants, or because different languages do not contain equivalents for some terms used in English, were the most frequent methodological and practical challenges identified by participants. In some circumstances, the employment of an interpreter to assist with communication may not be acceptable to participants, particularly in participants from some small communities and some refugee communities where confidentiality cannot be assured.

*Trust and engagement:* The next most frequently mentioned challenge was establishing trust and engaging communities and participants. It was suggested that researchers may need to first develop and build trust and credibility within the communities of interest for them to be accepted by these communities prior to undertaking any research. In the absence of trust and engagement with the community, the recruitment of participants from these communities was thought to be difficult or impossible.

*Limited resources:* The limited resources devoted to mental health and suicide research with people from migrant and refugee backgrounds was seen as both a methodological and practical challenge. This was thought to be particularly so because research in these areas and with these communities is particularly resource-intensive due to difficulties in recruitment, the need for interpreters and many other factors. It was also noted that the scarcity of mental health and suicide research with migrants impacts negatively on the quality of available data that researchers can use to inform their research design.

*Representation and safety:* Recruiting participants and being able to recruit a representative sample was identified as a methodological challenge. This was thought to be particularly challenging in work focusing on hard-to-reach groups and specific sub-groups with intersecting factors (e.g., LGBTIQA+ or persons living with disabilities) within particular communities. Overcoming stigma around mental health can also present methodological challenges for researchers. In communities where mental health issues and suicide are particularly stigmatized, there is a greatly reduced likelihood of engagement and participation. Potential participants, and the communities of which they are members, may feel that it is unsafe for them to participate, and that participation in the research and the findings of the research may negatively affect the participants’ or community’s wellbeing.

*Benefits to participants and communities:* Participants highlighted the importance of engaging with communities of interest to clearly identify and communicate the potential benefits of the research for the community and the measures in place to ensure that there would be no harm.

### 3.6. Ethical and Practical Challenges

*Different cultural expectations/values:* Negotiating different cultural values and expectations were seen as a key ethical challenge in conducting mental health and suicide research with migrant and refugee communities.

*Potential for harm and safety:* Identifying possible harms, putting in place clear measures to avoid such harms and to ensure safety, and communicating these matters during engagement with the community of interest were seen as crucially important components of ethically conducted research. This includes ensuring privacy and confidentiality, particularly when interpreters are required.

*Informed consent and confidentiality:* Obtaining fully informed consent was another challenge identified by the respondents. In the frequently encountered situation where the research consent procedures need to be translated for research participants who are not native English speakers, the accuracy of translation is something that must be ensured. The fact that many potential participants may not be familiar with research practices and how research is conducted is an additional challenge.

*Benefits to participants and communities:* As was noted in response to Question 3 on methodological and practical challenges, the need to ensure and effectively communicate that the research being undertaken is necessary and beneficial to the participants and the larger community as part of the process of engagement with the community of interest was highlighted. It was also suggested that the findings of the research should be disseminated and circulated for the use and benefit of the broader community.

*Ethics applications:* Some respondents noted that it is not infrequently challenging to obtain ethics committee approval for mental health and suicide research with migrant and refugee communities and that ethics committees need to be better informed about the need for and benefits of such research.

### 3.7. Strategies for Engaging Consumers and Carers

*Relationship-building and using networks:* It was suggested by participants that the primary strategy for engaging PWLE is to focus on relationship-building and using networks. The engagement of the community—for example, through influential community actors such as religious leaders, elders and other trusted and respected individuals within the community, as well as community-based service providers—at each stage of the research process was regarded as the most effective strategy. Actors such as community organizations can be useful channels through which connections with PWLE from migrant and refugee backgrounds can be established. The respondents also mentioned that researchers can also engage with community-based service providers, such as transcultural mental health centers, primary care doctors and school programs. Employing bilingual and multicultural workers from the relevant community was also seen as an important strategy as these individuals often already have established networks within their communities.

*Communication skills/strategies:* To overcome communication barriers, respondents recommended communication-centered strategies based on clarifying expectations and using culturally appropriate tools and questioning techniques. Being culturally aware and mindful of the individual, recognizing the lived experience of participants and gaining an understanding of their views on mental health and suicide were also deemed important for engagement. Employing bilingual and multicultural workers was also seen as an effective strategy.

*Developing skills:* Respondents recommended the development by researchers of skills and behaviors that demonstrate cultural sensitivity and competence as an important strategy for engaging with PWLE from migrant and refugee backgrounds. The availability of training and support for PWLE that enables them to develop their skills and knowledge of research was also seen as important. This also promotes trust and enables a comfortable and safe environment for PWLE where they can meaningfully contribute to research. Mentorship or peer support were seen as being valuable approaches to building research skills among PWLE.

*Value of research:* It was noted that if communities and potential participants cannot see the value and benefits of the proposed research, then participation would be substantially less likely.

*Empathy:* Values and behaviors such as empathy, kindness, humility, courtesy and honesty were noted to be important when approaching and engaging with PWLE from migrant and refugee backgrounds.

### 3.8. Strategies for Engaging Migrant Communities

There was a lot of overlap in the themes and recommendations concerning strategies for the engagement of PWLE and strategies for engagement of communities.

*Engagement:* Identifying and collaborating with community organizations and leaders was considered one of the most effective strategies to engage with the broader community on mental health and suicide research. Researchers may need to undertake preliminary work to locate and connect with trusted and respected community leaders who can facilitate connections with the broader community. Collaborating with community groups can also strengthen their capacity to respond to mental health and suicide issues within their communities. Researchers should also identify organizations that serve their communities and attend or organize community events such as consultations, forums, conferences, workshops, and public presentations to share knowledge with the broader migrant and refugee communities.

*Training and advocacy:* Several participants recommended the use of research methods that are engaging, participatory, and driven by the communities. Action research was identified as a research method that can be used to encourage the involvement of PWLE in mental health and suicide research as it allows communities to understand and see the practical implications and outcomes of the research. Furthermore, participatory research can also function as a form of training for PWLE and contribute to developing their own research knowledge and skills.

*Use of multiple media*: Researchers can use mainstream and ethnic print and electronic media and, increasingly, social media, to engage with the broader migrant and refugee communities.

## 4. Discussion

It is now increasingly recognized that migrants are underrepresented in mental health and suicide research (Carbone, 2021; Minas et al., 2007, 2013) and that such research in multicultural societies must be greatly expanded (State of Victoria, 2021; World Health Organization, 2023a). Without such expansion, the current evidence gaps will continue to perpetuate inadequate policy responses, inequitable access to effective mental health care and suicide prevention programs and poorer mental health outcomes for migrants (Minas et al., 2013). Further, it is understood that research with marginalized communities requires specific attention by researchers to issues of equity, diversity and inclusion. Ruzycki and Ahmed (Ruzycki & Ahmed, 2022) have suggested that a number of equity, diversity and inclusion principles must inform every aspect of the research process, in conceptualization, study design, conducting the research, data analysis and reporting and dissemination of the findings.

The results of this study are consistent with the principles enunciated by Ruzycki and Ahmed (Ruzycki & Ahmed, 2022) and re-affirm the numerous issues and challenges identified in health research with migrant and refugee populations over the past few decades, including the integrity of the consent process (Block et al., 2012; Sevimli, 2022), linguistic problems (Hoopman et al., 2009; Lee et al., 2014), community engagement (Casado et al., 2012; Holzer et al., 2014; McCabe et al., 2023; Pittaway et al., 2010), participatory methods (Desai et al., 2019; Guerin & Guerin, 2007) and representativeness (Enticott et al., 2017; Jacobsen & Landau, 2003).

The results highlight the key role of cultural and contextual considerations in mental health and suicide research. Like other groups, people of migrant and refugee backgrounds have varying conceptions of mental health, illness and mental health treatment and services and varying beliefs about and attitudes to suicide. As indicated by participants, researchers need to be aware of these cultural and contextual considerations to better understand how people from migrant and refugee backgrounds view mental health and suicide research, and their reluctance or readiness to participate in such research.

Language is a centrally important issue that presents a considerable challenge when undertaking research with migrants. Although working with interpreters, especially those known to the community, is seen as a strategy to engage with people from migrant and refugee backgrounds, this can also present challenges. As the respondents noted, methodological and ethical issues exist when employing interpreters who come from the same community as the participants. Although this is more likely to occur in small communities, researchers should be aware of its potential complications. Participants may hesitate to disclose information or refuse to participate when they personally know the interpreters because confidentiality and privacy may be compromised.

Key individuals such as community and religious leaders have been found to facilitate mental health service utilization (Colucci et al., 2012). Based on the results of this survey, organizations and individuals with leadership roles in their communities can also contribute to the better engagement of people from migrant and refugee populations in mental health and suicide research.

The personal characteristics of the researcher also play a role when undertaking research. Reflexivity, being open-minded and non-judgmental, and having an understanding of the similarities and differences between their experiences and those of migrants, are some facilitating factors identified in the literature (Pernice, 1994). Identifying qualities that can hinder participation or impact negatively on the research is also important (Pernice, 1994). Researchers should be prepared to have any preconceived ideas and understandings of the populations they intend to work with challenged. Researchers should be reflexive in their approach and have an understanding that some difficulties that they may encounter in the research process (e.g., relevance and appropriateness of research questions or methods) can also be the product of inaccurate cultural preconceptions.

As indicated by participants, trust between the researchers and the participants is a key element when undertaking research with migrant and refugee populations. Suspicion about the research and the motivations of the researcher, fear of authority and fear of possible harms from the research are some of the barriers to participation that have been identified in the literature (Guerin & Guerin, 2007; Pernice, 1994). To overcome these impediments, participatory methods and engagement with the populations over time are necessary (Guerin & Guerin, 2007).

Collaborative and participatory methods with the participants (Colucci & McDonough, 2020) ensure that the research reflects the sociocultural reality of the participants (Dean et al., 2012). The respondents recommended action research and other research methodologies that directly benefit the community as they engage the participants as well as educate them about the research process and contribute to developing research and dissemination skills.

Researchers must have the necessary knowledge and skills to design, conduct and communicate research in ways that reduce racism and other forms of discrimination and exclusion and that do not perpetuate health inequities and continuing marginalization of already marginalized population groups. This includes the skills required to engage with diverse populations, to build trust in the research process and to encourage participation by migrants and their communities in all stages of the research, from defining research questions, to study design and completion and the use of research results for the benefit of diverse communities.

## 5. Limitations and Strengths

The number of participants in this study was small and the study was carried out in Australia. The applicability of the findings beyond the Australian context is unknown. Similar studies in other migrant-receiving countries will be needed to determine whether similar knowledge and skills are required to conduct effective research with migrants in other contexts.

Despite efforts to recruit people with lived experience of mental health conditions, there were no participants with such lived experience among the survey respondents who identified as having expertise and experience in mental health and/or suicide research, which was an inclusion criterion for this component of the study. However, a strength of the study in terms of broader lived experience was that nearly half of the survey respondents were overseas-born and had the lived experience of being a migrant or refugee, as well as experience of carrying out research with migrants.

A further limitation is that the study investigated the views of experts concerning the required knowledge, skills and strategies. Whether the knowledge, skills and strategies that were identified by the experts in this study are in fact necessary and effective for mental health and suicide prevention research with migrants is a matter for further study.

As mentioned in the Methods Section the data reported here were collected in 2013. There are several reasons for our decision to publish data that were collected a decade ago.

The first is that it is almost universally acknowledged that carrying out effective clinical work with migrants requires specific cultural and related knowledge and skills (Kirmayer, 2012; Whaley & Davis, 2007). This has not been so in relation to mental health and suicide research with migrants. Very few studies have sought to identify the knowledge and skills required to effectively conduct such research.

The second is that virtually all the studies that have investigated one or more aspects of the issues examined here were published prior to 2013 (Block et al., 2012; Casado et al., 2012; Dean et al., 2012; Guerin & Guerin, 2007; Hoopman et al., 2009; Jacobsen & Landau, 2003; Pernice, 1994; Pittaway et al., 2010) with only a few more recent exceptions that have examined single issues, such as the recruitment of refugees for health research (Gabriel et al., 2017), informed consent (Sevimli, 2022) and digital story-telling (Colucci & McDonough, 2020). There has been no study published in the last decade that we have found that has attempted a comprehensive examination of the issues that are reported here.

The third, and most immediate and substantial, reason is that there is increasing recognition of the substantial gaps in the knowledge concerning mental health and suicide prevention services and programs for migrants, and corresponding calls for increased research to fill these gaps. The yawning knowledge gaps can only be filled by high-quality research if there are sufficient researchers who are aware of, and have developed, the necessary knowledge and skills to carry out such research with migrants and are able to engage migrants in such research.

A strength of the study is that a comprehensive examination of the necessary knowledge and skills has been carried out while most of the existing and still-limited amount of literature has focused on one or more aspects of the knowledge and skills required to conduct effective mental health and suicide research with migrants.

## 6. Conclusions

This paper adds to the existing knowledge by identifying the knowledge and skills required by researchers to carry out effective mental health and suicide research with migrant and refugee communities, identifying specific methodological, practical and ethical challenges in such research, and by suggesting practical strategies for the effective engagement of migrant and refugee communities in increasingly collaborative approaches to mental health and suicide research.

The findings from this project can be used to inform researchers on how to ethically and effectively undertake mental health and suicide research with migrant and refugee populations. They can also be used to develop research methods’ training modules (Ruzycki & Ahmed, 2022) that are specifically focused on carrying out research with migrant and refugee communities.

As mentioned in the Limitations Section, the knowledge, skills and strategies reported constitute the views of research experts. There is also a need to investigate the views of PWLE from migrant communities concerning the knowledge and skills that are required and the strategies that are likely to be successful in engaging migrant and refugee communities in mental health and suicide research. The absence of PWLE from the respondents in this study means that the reported findings should be regarded as provisional. Future investigations of the issues that are the subject of this study should be co-designed, carried out and reported with migrants, particularly PWLE, as partners in the research (Desai et al., 2019; Liem et al., 2024; Romios et al., 2008).

In addition, the question of whether these findings are applicable in contexts other than Australia where there are also large numbers of migrants can only be answered by the replication of this work in other countries and by recruiting international panels of experts.

## Figures and Tables

**Table 1:** Number and percent of total populations of migrants globally and in Australia: 2000–2020.

Year	Number of International Migrants Globally (Millions)	Migrants as a Proportion (%) of the World’s Population	Number of Migrants in Australia (Millions)	Migrants as a Proportion (%) of the Australian Population
2000	173	2.8	4.39	23
2005	192	2.9	4.88	24.2
2010	221	3.2	5.88	26.7
2015	248	3.4	6.73	28.3
2020	281	3.6	7.67	29.9

Sources: Global figures (McAuliffe & Oucho, 2024); Australian migration figures (Australian Bureau of Statistics, 2024).

**Table 2:** Expert panel members—primary areas of activity.

Primary Areas of Activity	Number	Percent
University or other research center	8	25.8
Government/policies	2	6.5
Mental health or suicide prevention service specific for refugees and/or migrants	9	29
Other mental health or suicide prevention service	10	32.3
Other service for refugees and/or migrants (non in mental health and suicide)	1	3.2
Other	1	3.2
Total	31	100

**Table 3:** Main emergent themes in response to each of the 6 questions.

Questions	Main Themes	Theme Mentions: Frequency	
		N *	%
**1. Required research skills and competencies**	1.1 Engagement skills	41	49.40%
Number of responses **: 75	1.2 Research skills, experience and qualifications	26	31.30%
	1.3 Communication skills	16	19.30%
**2. Required knowledge**	2.1 Knowledge of context	50	66.70%
Number of responses: 73	2.2 Research knowledge	18	24.00%
	2.3 Communication	7	9.30%
**3. Methodological and practical challenges**	3.1 Values and beliefs	18	22.50%
Number of responses: 75	3.2 Language difficulties	16	20.00%
	3.3 Trust	13	16.30%
	3.4 Engagement	12	15.00%
	3.5 Lack of resources	10	12.50%
	3.6 Representation	8	10.00%
	3.7 Safety	2	2.50%
	3.8 Benefit to participants/communities	1	1.30%
**4. Ethical challenges**	4.1 Different cultural expectations/values	21	28.80%
Number of responses: 68	4.2 Potential for harm, and safety	15	20.50%
	4.3 Informed consent and confidentiality	14	19.20%
	4.4 Benefit to participants and communities	14	19.20%
	4.5 Ethics applications	9	12.30%
**5. Strategies for engaging consumers and carers**	5.1 Relationship-building and using networks	27	34.20%
Number of responses: 66	5.2 Communication skills/strategies	26	32.90%
	5.3 Developing skills	13	16.50%
	5.4 Value of research	10	12.70%
	5.5 Empathy	3	2.80%
**6. Strategies for engaging migrant communities**	6.1 Engagement	44	57.10%
Number of responses: 67	6.2 Training and advocacy	15	19.50%
	6.3 Use of multiple media	18	23.40%

* The total number of theme mentions is often greater than the total number of responses because more than one theme was sometimes identified in a single response. ** Each respondent was invited to offer up to three responses to each question.

## Data Availability

The data presented in this study are available on request from the corresponding author.
